# Trauma-Informed Psychotherapy for Complex Post-traumatic Stress Disorder in a Young Adult Woman: A Case Report

**DOI:** 10.7759/cureus.105984

**Published:** 2026-03-27

**Authors:** Yibali N Shetty, Gunjan Y Trivedi, Gopal Bhatia

**Affiliations:** 1 Society for Energy and Emotions, Wellness Space, Ahmedabad, IND; 2 Psychiatry, Ahmedabad Psychiatry Clinic, Ahmedabad, IND

**Keywords:** childhood trauma, complex ptsd (cptsd), inner child integration therapy, self-harm, trauma-informed therapy

## Abstract

This case study, conducted at a wellness center in western India, examines the therapeutic journey of a female in her early 20s with severe anxiety, depression, insomnia, self-harm urges, suicidal ideation, and a history of childhood trauma. Standardized assessments, including the Generalized Anxiety Disorder-7 (GAD-7), Major Depression Inventory (MDI), insomnia assessment, Adverse Childhood Experiences (ACE-14), Suicide Behaviors Questionnaire-Revised (SBQ-R), and the International Trauma Questionnaire (ITQ), revealed severe symptoms and the presence of complex post-traumatic stress disorder (CPTSD).

A detailed consultation identified additional issues not fully captured by structured assessments, including a history of self-harm, suicidal thoughts, a chronically violent and abusive family environment, dysfunctional coping strategies, irrational decisions, attachment disturbances, and instability in relationships. A trauma-informed, integrative psychotherapeutic approach was implemented over more than one year, incorporating inner child therapy, parts work, empty chair and Gestalt-based body interventions, self-regulation practices, and belief system restructuring, followed by later-stage coaching interventions. Outcomes demonstrated substantial improvements in emotional regulation, trauma resolution, relationships, and resilience.

This case highlights the importance of combining structured CPTSD assessments with detailed interviews and phased trauma-informed psychotherapy.

## Introduction

Complex post-traumatic stress disorder (CPTSD), as conceptualized in ICD-11, is associated with a history of prolonged or repeated exposure to trauma, often occurring during childhood [1].

Post-traumatic stress disorder (PTSD) is typically characterized by three core symptom clusters: (1) re-experiencing, (2) avoidance, and (3) a persistent sense of threat (often seen as hyperarousal/hypervigilance). CPTSD, in addition to the three core PTSD symptoms, includes affective dysregulation, negative self-concept, and relational disturbances, collectively referred to as Disturbances in Self-Organization (DSO) [1]. Individuals with CPTSD frequently present with self-harm behaviors, abandonment issues, attachment issues, affective instability, addiction, irrational decision-making, and dysfunctional coping strategies [2]. Although the DSM-5 does not currently include a specific CPTSD diagnosis, recent studies estimate that CPTSD accounts for nearly 50% of trauma-related presentations in mental health centers [1]. Therefore, a greater understanding of CPTSD is needed among mental health practitioners, given its novelty and the unique assessment criteria for DSO components identified using the International Trauma Questionnaire (ITQ) [1,3,4].

The single-case study illustrates the assessment, formulation, and therapeutic process of an integrative, trauma-informed psychotherapy intervention in a young adult female with CPTSD symptoms, highlighting the role of a client-centric approach, regular assessments, and phased treatment [5,6].

## Case presentation

The individual, a single female in her early 20s, signed informed consent and sought help at a wellness center in western India with presenting symptoms of severe psychological distress and impaired functioning across emotional, relational, and behavioral domains in August 2024. These symptoms had existed for more than 12 months at the time of presentation. Initial consultation identified repeated exposure to childhood trauma, assessed during a personal interview using the India-specific Adverse Childhood Experiences (ACE) assessment with 14 questions [7]. The ACE questionnaire evaluates exposure to potentially traumatic experiences before the age of 18. The ACE framework assigns one point for each affirmative response. Previous research has identified a dose-response relationship between exposure to ACEs and mental health conditions, indicating that higher exposure to ACEs poses a higher risk for mental health conditions [8,9]. In the present case, the individual had an ACE score of 8, highlighting very high exposure to childhood trauma. Reported experiences included very frequent emotional abuse and emotional neglect, occasional sexual abuse, intermittent physical abuse, frequent peer rejection, recurrent exposure to parental verbal fights, addiction within the household, and concerns regarding a parent’s mental health condition.

The specific ACE domains reported by the individual are described as follows: (1) Emotional abuse: She reported chronic emotional abuse from her father, characterized by prolonged silent treatment, emotional unavailability, and persistent criticism despite good academic performance. She described little emotional bonding between them. (2) Physical abuse: She recalled intermittent physical punishment from her mother, primarily related to her academics. (3) Sexual abuse: She reported multiple instances of sexual abuse (inappropriate touch) by a neighbor of similar age for a few years during her early developmental years, starting at around six years of age. (4) Emotional neglect: She experienced significant emotional neglect within the multigenerational household. She consistently experienced a lack of physical affection, validation, and emotional bonding within the family system. She felt unseen, unheard, and unimportant, and often sought repeated validation and approval, especially from her father. Although her grandparents lived in the same home, she felt emotionally distant from them and lacked warmth. (5) Peer isolation and rejection: While growing up, she had trouble forming and maintaining friendships. She often attempted to gain acceptance through excessive accommodation and people-pleasing behaviors. Over time, this pattern became emotionally exhausting, leading her to withdraw from peer groups. She frequently interpreted these experiences as confirmation that she did not belong, even though she did not recall explicit rejection from peers. (6) Parental fights: She frequently witnessed intense verbal conflicts between her parents, and she also recalled several instances of verbal conflicts between her parents and grandparents. (7) Addiction in the household: Her grandfather had alcohol dependence, which she frequently witnessed while growing up. (8) Parental mental health: She perceived her father as being depressed, although no formal diagnosis or treatment was present.

Further consultation revealed a history of non-suicidal self-injury (NSSI), suicidal ideation, strained family relationships, and substance use. She had also previously received psychiatric medications, which were gradually tapered down under psychiatrist supervision. She also felt emotional numbing due to those medications, and she was not on any medications during the initial consultation.

She reported that she first engaged in self-harm by cutting herself at approximately 15 years of age. She described the behavior as a coping mechanism during periods of overwhelming emotional distress. Episodes were often associated with feelings of inadequacy, guilt, and a persistent sense of failing to meet familial expectations. She frequently felt that she was not a good daughter and suppressed her emotions, which intensified the internal distress. Self-harm (cutting wrists and thighs) provided temporary emotional relief from these emotions, and over time, the behavior became habitual. While NSSI did not have the intention to take her life, there was a phase of her life where she used to have suicidal thoughts, especially during the days preceding her first visit.

During childhood, she reported being strongly attached to her father and frequently sought his validation. To gain his approval, she discontinued interests such as dancing and singing after he expressed disapproval. Instead, she adopted activities she believed would earn his approval, such as reading newspapers and playing chess. Repeated experiences of emotional unresponsiveness, silent treatments, criticism, and strict disciplinary practices contributed to her developing people-pleasing tendencies and internalization of guilt and self-blame. This gradually led to a significantly strained relationship with her father. Although she mentioned she was not close to her mother during childhood, she currently has a supportive relationship with her.

Her paternal grandparents lived in the same household while she was growing up. The family environment during childhood was described as chaotic, with frequent verbal altercations between parents and ongoing intergenerational conflicts. She often perceived her mother as frequently cornered during these conflicts, and subsequently developed a protective attitude towards her. These experiences contributed to longstanding feelings of loneliness, emotional invalidation, and a perception of not meeting familial expectations.

The individual reported that she entered her first romantic relationship at the age of 14, which was strongly disapproved of by her parents, particularly her father, and resulted in verbal criticism and humiliation. Despite the difficulties, she remained in the relationship for some time; it eventually ended. At around 15 years of age, she began another relationship with a supportive childhood friend, which lasted approximately two years. The relationship ended when her partner relocated for educational purposes. She described the breakup as distressing and reported engaging in brief, casual relationships and increased smoking as coping mechanisms.

Despite emotional challenges, she maintained strong academic performance throughout childhood. In the ninth grade, despite achieving academic excellence, her father did not acknowledge her accomplishments. The following year, she performed well, but attention focused on the marks she did not achieve rather than her success. As a result, she developed a pattern of using academic achievement to seek validation from her parents. Although she reported the ability to temporarily suppress emotional stress to maintain her academic performance, this coping pattern was associated with considerable internal pressure and fear of failure.

The client also described a disrupted relationship with food, reporting that she rarely experienced hunger and often avoided eating during periods of emotional distress. At other times, meals were consumed more out of routine than appetite, because food was prepared at home. Over the course of therapy, and with the introduction of self-regulation practices, she gradually began to experience natural hunger cues and developed a more regular eating pattern. Eventually, she was able to consistently have three meals a day.

She began smoking cigarettes and occasionally consuming alcohol at around 17 years of age, primarily in social settings with peers. She did not enjoy alcohol, but continued smoking. After a relationship breakup, cigarette use increased from occasional experimentation to approximately two cigarettes per day. Later, at the age of 20, she had to move to another city to pursue a new academic direction. This transition was associated with increased stress related to studies and social adjustment, during which cigarette consumption gradually escalated to approximately 20 cigarettes per day. She began noticing negative consequences, including reduced physical stamina and financial strain, which motivated her to address the behavior during therapy.

She first engaged in psychological counselling at the age of 16. After some time, she transitioned to another psychologist; however, she experienced only temporary improvement and eventually discontinued therapy. At the age of 19, following a significant romantic breakup, she sought psychiatric consultation in November 2023. She was diagnosed with impulse control disorder with depressive episodes and was prescribed psychotropic medications, including oxcarbazepine (anticonvulsant/mood stabilizer), aripiprazole (atypical antipsychotic), and lithium carbonate (Carbolith). She was also on cilnidipine for hypertension. During treatment, she maintained regular follow-up visits for approximately two months. In January 2024, the psychiatrist advised discontinuation of pharmacological treatment and recommended psychotherapy.

In August 2024, the individual presented to our wellness center seeking psychological support. Prior to initiating therapy, suicide risk was assessed using the Suicide Behaviors Questionnaire-Revised (SBQ-R). She obtained a score of 12, indicating a high risk for suicidal ideation. According to the center’s clinical protocol, individuals scoring ≥7 on the SBQ-R are referred for psychiatric evaluation to confirm readiness for psychotherapy [9-13]. The psychiatrist identified attachment-related difficulties, trust issues, and fear of abandonment as central concerns and recommended that she proceed with psychotherapy without pharmacological intervention.

Her self-assessments, prior to consultation, highlighted severe levels of anxiety, depression, and insomnia, as shown in Table 1 [12-14].

**Table 1:** Assessments of WHO-5, MDI, GAD-7, and ISI before and after therapeutic intervention WHO-5: World Health Organization-Five Well-Being Index: GAD-7: Generalized Anxiety Disorder-7; MDI: Major Depression Inventory; ISI: Insomnia Severity Index

Timing	Well-being (WHO-5: Well-Being Index) - Desired > 52	Anxiety (GAD-7: Generalized Anxiety Disorder) - Desired < 9	Depression (MDI: Major Depression Inventory) - Desired < 19	Insomnia (ISI: Insomnia Severity Index) - Desired < 9
Before consultation	4	18	43	23
After 6 sessions	24	14	35	24
After 12 sessions	16	15	37	20
After 16 sessions	68	4	11	10
After 30 sessions	80	2	5	9

All the tools for assessment used in the case report - WHO-5 (Well-Being Index) for well-being, GAD-7 (Generalized Anxiety Disorder) for anxiety, MDI (Major Depression Inventory) for depression, ISI (Insomnia Severity Index) for insomnia, ITQ, and BRS (Brief Resilience Scale) - were used. They are widely used, validated, and freely accessible measures in psychological research and clinical practice [4-17].

Given the individual’s history of repeated exposure to trauma, self-harm behavior, and suicidal ideation, assessment for CPTSD was conducted using the ITQ [4]. The ITQ assesses symptoms of PTSD, DSO, and CPTSD. Items are rated on a scale from 0 (Not at all) to 4 (Extremely); higher scores indicate greater symptom severity. For interpretation, a probable PTSD diagnosis requires endorsement (score ≥2) of at least one symptom in each PTSD cluster (re-experiencing, avoidance, sense of threat), along with associated functional impairment. A CPTSD diagnosis requires that PTSD criteria are met, in addition to endorsement (≥2) of symptoms across all three DSO domains (affective dysregulation, negative self-concept, relational disturbance), along with functional impairment.

In the present case, the individual obtained a PTSD score of 19 and a DSO score of 19, meeting criteria for CPTSD. The PTSD score reflected moderate to high severity of core trauma symptoms, including recurrent re-experiencing, avoidance of trauma-related reminders, and a persistent sense of threat, such as hypervigilance. The equally elevated DSO score indicates substantial DSO. These were largely driven by a high negative self-concept score of 7 and an affective dysregulation score of 7, followed by a disturbed relationships score of 5, suggesting pervasive shame or worthlessness, alongside significant emotional instability and difficulties in interpersonal trust and closeness, consistent with the broader and more impairing profile of CPTSD.

Psychological resilience was assessed using the BRS, which measures an individual’s ability to recover from stress. Scores range from 1 to 5, where 1.00-2.99 indicates low resilience, 3.00-4.30 reflects normal resilience, and 4.31-5.00 indicates high resilience, with higher scores indicating a greater capacity to bounce back from adversity [16,18]. In this case, the client’s resilience improved from 1.3 before therapy (low resilience) to 3.2 after 12 sessions of treatment (within the normal range), and further increased to 4.3 by the end of 30 sessions, reflecting a marked improvement in coping capacity and psychological adaptability.

The intervention followed an integrated, trauma-informed therapeutic approach over a span of one year and comprised 30 sessions. The therapeutic process involved a trained therapist, regular progress reviews by a psychologist, and periodic consultations with a psychiatrist. During the initial phase, sessions were conducted weekly for approximately 12 weeks, followed by biweekly sessions for several months, and later tapered to monthly sessions as the client’s emotional stability improved. Although the intensity of sessions gradually reduced, therapy was not discontinued entirely, and follow-up sessions were scheduled approximately once every two months, or as needed, based on the client’s emotional needs. The early sessions focused on personalized training in self-regulation techniques, including the self-hypnosis protocol, guided imagery (sleep script), and emotional freedom technique (EFT), which helped the client manage distress, regulate emotional responses between sessions, and improve sleep quality. A structured self-hypnosis protocol developed by the Society of Energy and Emotions was taught as a daily practice [19,20]. This routine involved four sequential steps, practiced for approximately five minutes each. The process began with humming to calm the nervous system, followed by coherent breathing aimed at synchronizing the heart rate with the breathing rate. The third step involved consciously invoking positive emotions to reconnect with her inner strengths. The final step included guided imagery and personalized affirmations, allowing her to visualize a desired future self, and also repeat the affirmations she had written for herself.

EFT was also introduced to help her process distressing emotions. She was guided to tap on specific acupressure points while acknowledging the emotional experience. Each round of tapping was followed by brief eye movements, humming, and seven slow, deep breaths, which together were intended to support emotional release and reduce physiological arousal.

Given her sleep issues, a personalized sleep audio script was provided. This included progressive muscle relaxation, guided imagery, and affirmations designed to quiet the mind and body at bedtime, gradually improving sleep quality [21].

In addition, grounding strategies, such as the 5-4-3-2-1 sensory technique, were introduced to help her anchor to the present moment during periods of overwhelm. Simple deep-breathing exercises and bringing her awareness to her breath were encouraged to help regulate her emotions throughout the day. Together, these practices helped reduce excessive sympathetic nervous system activation while strengthening parasympathetic regulation, thereby promoting autonomic balance and emotional stability.

Once a degree of emotional stabilization was achieved and rapport built, therapy progressed toward processing key trauma experiences, particularly those connected to the client’s relationship with her father. Inner child work was used to process unresolved developmental experiences related to emotional abuse, emotional neglect, inappropriate touch, and episodes of silent treatment by the father [5,22]. Through guided imagery and therapeutic dialogue, the client was encouraged to reconnect with vulnerable child states in a safe, compassionate manner. Emphasis was placed on validating unmet developmental needs and fostering a nurturing internal dialogue through therapeutic reparenting.

Parts work was employed to explore internal conflicts and coping patterns [23]. She identified several internal parts that had emerged in response to earlier experiences, including a protective part, a manipulative part, a people-pleaser part, a smoking part (“Energy Gainer”), and a non-smoking part (“The Change”). Therapy guided her to gently recognize and understand these parts, appreciate the costs and intentions behind their behaviors, and facilitate dialogue between them, with the goal of integrating the parts in a way that would help the client feel whole. This approach promoted integration, self-acceptance, and improved emotional regulation.

Experiential techniques derived from Gestalt therapy, developed by Fritz Perls, were also integrated into the therapeutic process [24]. Gestalt-oriented body work emphasizes awareness of bodily sensations, posture, and breathing as a means of accessing emotional experiences that may remain unprocessed cognitively. During sessions involving emotionally charged memories, the client was encouraged to express suppressed emotions through embodied expression, such as crying into a pillow or vocalizing emotions while bringing awareness to areas of bodily tension, breathing patterns, and posture. This process helped facilitate emotional release and reconnection with bodily awareness. The Gestalt Empty Chair technique was also used to support emotional processing of unresolved relational experiences. Through imagined dialogue with significant individuals or disowned aspects of the self, the client was able to express previously unspoken feelings and work through unfinished emotional experiences. Empirical evidence supports the efficacy of chair-based experiential methods in improving emotional regulation, reducing somatic distress, and enhancing internal coherence.

Psychoeducation formed an important component of therapy and included developing an understanding of the psychological and physiological impact of trauma, the mechanisms underlying CPTSD, and common survival responses such as fight, flight, freeze, and fawn. The client was also educated about the role of the autonomic nervous system in trauma responses and the difference between explicit (conscious) and implicit (body-based) traumatic memories, with emphasis on addressing implicit memories to reduce persistent distress. Additional psychoeducation included understanding the locus of control, recognizing relational dynamics such as the Karpman drama triangle within family systems, identifying activities that either drained or replenished her energy, and maintaining a reflective journal to enhance emotional awareness.

During therapy, when the client had academic examinations, guided imagery using hypnosis was introduced to help her approach exam situations with greater calmness and focus. These sessions involved visualizing herself preparing for and completing examinations with confidence. Hypnotic anchoring techniques were used to strengthen confidence and self-esteem so that these emotional states could be accessed in stressful situations. After processing key trauma events during the initial phase of therapy, a reduction in disturbance in the DSO symptoms was observed (Table 2).

**Table 2:** Trauma assessments before and after therapeutic intervention PTSD/DSO/CPTSD were assessed using the ITQ. PTSD: Post-traumatic Stress Disorder; CPTSD: Complex PTSD; DSO: Disturbance in Self-Organization; ITQ: International Trauma Questionnaire

Timing	Diagnosis	PTSD Score	DSO Score	CPTSD Score
Before consultation	CPTSD	19	19	38
After 16 sessions	PTSD	7	3	10
After 30 sessions	None	2	0	2

As the client’s emotional stability improved, therapy gradually transitioned into a coaching-oriented phase focused on life direction, autonomy, and personal development. Coaching principles, informed by the framework described in "The 7 Habits of Highly Effective People" by Stephen R. Covey, were incorporated to support value-based decision-making and long-term goal alignment [25].

A structured session using the Life Priority Matrix (to help her prioritize key areas of life, such as emotional health, relationships, etc.) was periodically used to help the client evaluate satisfaction across different life domains and compare it with the long-term importance she attributed to them. She ranked the different life domains and assessed the discrepancy between perceived importance and current satisfaction. Identified gaps were prioritized, and stepwise action plans were developed to address these areas, which helped in the development of smart, practical goals aligned with her personal values.

Guided imagery was used for visualizing the future self, to help the client reflect on the kind of life, relationships, and personal qualities she wished to cultivate.

During therapy, the client demonstrated increasing emotional awareness and autonomy. She was able to communicate her academic interests more openly with her parents and chose to pursue psychology as a field of study, reflecting greater alignment with her personal aspirations.

This transition required relocating to another city and presented new challenges, during which she reported an increase in smoking behavior. The concern was subsequently addressed using parts work, facilitated through hypnosis, allowing dialogue between the smoking and non-smoking aspects of self. A personalized audio script, incorporating agreed-upon resolutions, future pacing, and post-hypnotic suggestions, was provided to reinforce behavioral change and support a shift from compulsive smoking toward more conscious and choice-based behavior.

Across the 30 sessions, the client demonstrated substantial improvements in anxiety, depressive symptoms, sleep quality, emotional regulation, and overall psychological well-being. Self-harm urges, which had been present earlier in therapy, gradually reduced over time and were completely absent by the end. Interpersonal functioning also improved, particularly in her relationship with her parents; she reported healthier communication and an increased ability to establish and maintain personal boundaries. Alongside symptom reduction, the client demonstrated greater emotional awareness, improved coping strategies, and increased autonomy in decision-making.

The observed reductions in trauma-related symptoms, together with the improvement in resilience, indicate meaningful clinical progress. The transition from diagnostic criteria consistent with complex trauma at baseline to the absence of diagnostic criteria by the end of therapy suggests substantial recovery in both trauma symptoms and disturbance in self-organization (Table 2). These changes were accompanied by improvements in emotional regulation, interpersonal functioning, and overall psychological well-being.

## Discussion

The present case illustrates the profound psychological impact of cumulative ACE and their association with symptoms consistent with CPTSD. As described in ICD-11, CPTSD develops following prolonged or repeated interpersonal trauma and is characterized not only by core PTSD symptoms but also by DSO. The client in this case reported multiple forms of childhood adversity, which appeared to contribute to persistent emotional distress, interpersonal difficulties, and maladaptive coping behaviors, such as self-harm and smoking during early adulthood.

The client’s ACE score of 8 reflects a high level of cumulative childhood adversity. Previous research has consistently demonstrated a dose-response relationship between ACE exposure and later mental health outcomes, including depression, anxiety, substance use, and suicidal behavior. Experiences of chronic emotional invalidation and relational instability during childhood appear to contribute to persistent feelings of inadequacy, people-pleasing tendencies, and fear of abandonment, which are commonly observed in individuals with complex trauma histories.

In this particular case, the client reached out for psychotherapy in August 2024 after her psychiatrist stopped medications in January 2024. Our team also consulted the collaborating psychiatrist to validate the approach of pursuing psychotherapy without medications. Assessment using the ITQ confirmed the presence of both PTSD and DSO symptoms, supporting a probable diagnosis of CPTSD, with elevated scores across affective dysregulation, negative self-concept, and relational disturbance. Over the course of therapy, substantial reductions in trauma-related symptoms were observed, and by the end of treatment, the client no longer met criteria for CPTSD. Consistent improvements were observed across symptom domains, including anxiety, depression, sleep, and overall psychological well-being, along with a significant increase in resilience, as measured by the BRS.

The therapeutic approach in this case followed a trauma-informed and integrative framework. The initial phase of treatment prioritized emotional stabilization and the development of self-regulation skills through practices such as self-hypnosis protocol, EFT, breathing exercises, grounding exercises, and guided imagery for sleep (sleep script). These interventions are known to support the regulation of autonomic nervous system functioning, which is often dysregulated in individuals exposed to chronic trauma. Once emotional safety and therapeutic rapport were established, therapy progressed toward trauma processing through approaches including inner child work, parts work, and experiential Gestalt techniques, such as empty-chair and somatic release. These approaches facilitated the processing of unresolved developmental experiences while supporting the integration of fragmented aspects of self.

From a case formulation perspective, the client’s presentation can be understood using a 4P framework. Predisposing factors included repeated exposure to childhood adversity, emotional neglect, and patterns suggestive of insecure attachment within the family environment. Precipitating factors included stressful life events during adolescence and early childhood, particularly romantic relationship disruptions and academic transitions, that appeared to intensify existing emotional vulnerabilities. Perpetuating factors included maladaptive coping strategies, such as self-harm, smoking, emotional suppression, and persistent negative self-beliefs. Protective factors included the client’s academic strengths, motivation for self-improvement, engagement in therapy, and the gradual development of healthier coping strategies and interpersonal boundaries. Addressing these interacting factors through a structured therapeutic process likely contributed to the improvements observed.

There is limited work on CPTSD case studies. While several studies have explored CPTSD assessment, research, and case studies on how to address CPTSD remain limited [6,26-28]. The current case study adds to the limited knowledge base of intervention-based work on complex trauma, not just in India, but globally. Recent work on addressing CPTSD has focused on (a) event-specific work, using the concept of index trauma, and (b) intervention techniques such as reconsolidation of traumatic memories (RTM) and eye movement desensitization and reprocessing (EMDR) [28-31]. Given the limited prior research on CPTSD interventions, this case offers important clinical insights. First, a comprehensive assessment using multiple validated tools, along with a detailed clinical consultation, was essential to understanding the complexity of the client’s presentation. While the ITQ helped identify CPTSD symptoms, additional assessments evaluating anxiety, depression, sleep disturbance, resilience, and suicide risk provided a broader understanding of the client’s functioning. Importantly, the clinical interview played a crucial role in uncovering several developmental experiences, relational dynamics, and behavioral patterns that were not fully captured through standardized assessment tools. This highlights the importance of integrating structured assessments with in-depth clinical exploration when working with individuals presenting complex trauma histories. Second, routine suicide risk screening played an important role in ensuring appropriate clinical decision-making and early safety planning. Third, collaboration among the psychotherapist, psychologist, and psychiatrist contributed to a more comprehensive understanding of the client’s difficulties and supported an integrated treatment approach. Finally, this case highlights the importance of considering CPTSD in individuals presenting with multiple developmental trauma risk factors, even though it is not currently included in the DSM-5 diagnostic framework. Psychoeducation regarding the nature of complex trauma, the need for long-term engagement, and the role of patience in recovery appeared to support sustained participation in therapy.

In the later stages of therapy, coaching-oriented strategies were incorporated to support the client’s transition from symptom management to personal growth. As she began to stabilize emotionally, the focus gradually shifted toward helping her reconnect with her own preferences, values, and long-term goals. Through this process, she developed greater clarity about her academic direction and was confident in expressing her choices to her family. She demonstrated an increased ability to communicate her needs assertively, remain firm with her decisions, and draw healthy boundaries for herself. Alongside this, she developed healthier coping mechanisms and showed increased awareness of the impact of smoking on her physical health, which motivated her to actively work toward reducing it. This phase of therapy appeared to strengthen her sense of autonomy, self-trust, and identity, reflecting an important shift from external validation toward internal alignment.

This case report is limited by its single-subject design, absence of a control condition, and reliance primarily on self-report measures (except for the therapist-administered ITQ assessment). Future work should explore the risk factors for CPTSD and specific techniques.

## Conclusions

This case highlights the complex and long-term psychological impact of cumulative ACE and their association with symptoms consistent with CPTSD. It underscores the importance of incorporating CPTSD-focused assessment, particularly in individuals presenting with multiple childhood trauma risk factors, even in settings where it is not formally recognized within diagnostic frameworks such as the DSM-5. The findings emphasize the value of a comprehensive, multi-layered assessment approach, integrating standardized tools with in-depth clinical interviews to capture aspects of the client’s experience that may not be reflected in structured measures alone. Routine suicide risk screening and collaboration with psychiatric services further contributed to safe and informed clinical decision-making.

This case also demonstrates the effectiveness of an integrative, trauma-informed therapeutic approach that progresses from stabilization and self-regulation to trauma processing, and ultimately toward personal growth. The inclusion of coaching-oriented strategies supported the development of autonomy, improved decision-making, and the client’s ability to assertively communicate her needs, reflecting meaningful functional recovery beyond symptom reduction. Overall, the case highlights the importance of psychoeducation, a long-term therapeutic perspective, and a client-centered, flexible approach in supporting recovery from complex trauma.
